# Observations on the Diseases Incident to Pregnancy and Childbed

**Published:** 1840-10

**Authors:** 


### Art. XVIII.

*Observations on the Diseases incident to Pregnancy and Childbed.* By Fleetwood Churchill, m.d. &c.—*London*, 1840. 8vo, pp. 455.

Although we would not award to the best compilation the same meed of approbation that we would bestow upon a work of originality, we are inclined to regard the labours of an able compiler with much more respect than is by many thought to be his due. No man can make a good compilation who does not possess a perfect knowledge of the subject; a perspicuous style of writing; the tact of discriminating fact from fancy; and, lastly, the talent, which belongs to few, of patient and sustained industry. On the other hand, we confess, that we are inclined to measure out our praise with a sparing hand, to him who throws together the heterogeneous opinions of conflicting writers; but who, either from the consciousness of incapacity, or the dread of the labour which the task demands, omits to give a condensed and clear statement of the sum and substance of the best opinions up to the time he writes. The work before us is evidently, and indeed confessedly, a compilation, and we cannot assign to it the first rank among works of this sort. Every page shows the number of works which the author has consulted, but the materials derived from them are not, we think, always judiciously employed. We object to the custom of loading almost every page with numerous references to various authors, merely for the purpose of extracting commonplace statements, which are to be found in every elementary work, and which the writers never could have thought of claiming as their peculiar property. Dr. Churchill's book would, in our opinion, have been much more agreeable to read if he had avoided this fault; if he had made less show of research, and had confined his foot notes to the original opinions of the authors he quotes, and had given in his own language a condensed and unbroken statement of practical facts, that are established beyond dispute, and which every writer upon the same subject admits and states. If Dr. Churchill had adopted this plan, his very numerous foot notes would have been greatly diminished in number, but the well-instructed reader would not so frequently have had the question obtrude itself, of "why a foot note," to reiterate a statement in the text, and to refer to an individual authority for an opinion, which is to be found in almost every writer upon the subject during the last century.

Although, however, we cannot approve of the arrangement of this work, we freely and with pleasure admit that it contains a great deal of practical information on the diseases of pregnancy and childbed, which the student will peruse with advantage, and which certainly cannot be found in any one English work on the same subject. To those, too, who are inclined to "read up" the best opinions upon these practically important topics, the various and well-selected authorities from French, German, and English writers, quoted by Dr. Churchill, will be found very useful in lightening the labour of literary research.

